# Complete Genome Sequence of *Neisseria meningitidis* Serogroup A Strain NMA510612, Isolated from a Patient with Bacterial Meningitis in China

**DOI:** 10.1128/genomeA.00360-14

**Published:** 2014-05-08

**Authors:** Yan Zhang, Jian Yang, Li Xu, Yafang Zhu, Bo Liu, Zhujun Shao, Xiaobing Zhang, Qi Jin

**Affiliations:** a MOH Key Laboratory of Systems Biology of Pathogens, Institute of Pathogen Biology, Chinese Academy of Medical Sciences and Peking Union Medical College, Beijing, China; b State Key Laboratory for Infectious Disease Prevention and Control and the National Institute for Communicable Disease Control and Prevention, Chinese Center for Disease Control and Prevention, Beijing, China

## Abstract

Serogroup A meningococcal strains have been involved in several pandemics and a series of epidemics worldwide in the past. Determination of the genome sequence of the prevalent genotype strain will help us understand the genetic background of the evolutionary and epidemiological properties of these bacteria. We sequenced the complete genome of *Neisseria meningitidis* NMA510612, a clinical isolate from a patient with meningococcal meningitis.

## GENOME ANNOUNCEMENT

*Neisseria meningitidis* is a leading cause of bacterial meningitis worldwide (1). Among its 6 serogroups (A, B, C, X, Y, and W135) involved in invasive meningococcal disease, serogroup A strains caused pandemic waves and large epidemics in China and Africa during the last century (2). Interestingly, most of the epidemics before the 1990s were associated with a clone of subgroup III, called sequence type 5 (ST5) (3, 4), whereas replacement of ST7 strains occurred thereafter in both China and the African meningitis belt (5). Molecular genotyping of organisms involved in epidemics showed that ST7 strains with P1.20,9 represented over 86% of isolates in China after 2000 (6). Herein, we present the complete genome sequence of serogroup A strain NMA510612, a type strain of the ST7 genotype, which was isolated in China in 2006 from a patient with acute bacterial meningitis. The genome of NMA510612 will further our understanding of the molecular evolution of the hyperinvasive lineage of *Neisseria meningitidis*.

The NMA510612 strain was grown in a chocolate plate incubated overnight in 5% CO_2_ at 37°C. The chromosome DNA extraction was performed according to the protocol provided with the DNA extraction kit (Promega, Madison, WI, USA). For *de novo* sequencing, a fragment library with an average length of 500 bp was constructed and then sequenced with a Roche/454 GS FLX Titanium instrument (Roche Diagnostics, Penzberg, Germany). The raw data set of 219,392 reads (126 Mb) was assembled using Newbler version 2.6 (Roche), which produced 106 large contigs (>500 bp) with an average size of 19 kb. Then, gaps between these contigs were closed by genomic PCR and primer walking methods with conventional Sanger sequencing (7). Glimmer3 and tRNAscan-SE were used to predict protein-coding sequences (CDS) and tRNA genes, respectively. Annotation of the CDS was performed with BLAST searches of the GenBank nonredundant protein database followed by manual curation using Z2491 as a reference (GenBank accession no. AL157959.1) (8).

The complete sequence of the NMA510612 genome consists of one circular chromosome with a size of 2,188,020 bp, and the average G+C content is 51.5%. The chromosome is predicted to possess 4 rRNA operons, 163 insertion elements (IS), 59 tRNAs, and 2,462 CDS. The NMA510612 genome was demonstrated to be highly collinear with that of WUE2594 (GenBank accession no. FR774048), a strain of the ST5 genotype isolated from Germany in 1991 (9). The NMA510612 genome is smaller than the WUE2594 genome due to lack of a 42-kb Mu-like prophage region. On the other hand, it specifically harbors several genes, such as *tetA* and *hmbR*. Previous studies have demonstrated the presence of tetracycline resistance determinants like *tet*(M) and *tet*(B) (10, 11). The distribution of the hemoglobin receptor gene *hmbR* was investigated and observed at a significantly higher frequency among disease isolates than among carriage isolates (12). In addition, we found that polymorphic regions exist in genes encoding type IV pilus proteins and type I restriction enzymes. These variations may be involved in a rearrangement of surface-exposed proteins in this ST7 clone and enable the bacteria to overcome the herd immunity generated by the presence of the ST5 strain in the population.

### Nucleotide sequence accession number.

The genome sequence of *N. meningitidis* NMA510612 has been deposited in GenBank under accession no. CP007524.
